# Electronic Health Records–Based Cardio-Oncology Registry for Care Gap Identification and Pragmatic Research: Procedure and Observational Study

**DOI:** 10.2196/22296

**Published:** 2021-05-12

**Authors:** Alvin Chandra, Steven T Philips, Ambarish Pandey, Mujeeb Basit, Vaishnavi Kannan, Evan J Sara, Sandeep R Das, Simon J C Lee, Barbara Haley, DuWayne L Willett, Vlad G Zaha

**Affiliations:** 1 Cardiology Division Department of Internal Medicine University of Texas Southwestern Medical Center Dallas, TX United States; 2 Harold C Simmons Comprehensive Cancer Center University of Texas Southwestern Medical Center Dallas, TX United States; 3 Parkland Health & Hospital System Dallas, TX United States; 4 Clinical Informatics Center University of Texas Southwestern Medical Center Dallas, TX United States; 5 Department of Population and Data Sciences University of Texas Southwestern Medical Center Dallas, TX United States; 6 Hematology and Oncology Division Department of Internal Medicine University of Texas Southwestern Medical Center Dallas, TX United States

**Keywords:** electronic health records, cardio-oncology, patient registry, heart failure, screening

## Abstract

**Background:**

Professional society guidelines are emerging for cardiovascular care in cancer patients. However, it is not yet clear how effectively the cancer survivor population is screened and treated for cardiomyopathy in contemporary clinical practice. As electronic health records (EHRs) are now widely used in clinical practice, we tested the hypothesis that an EHR-based cardio-oncology registry can address these questions.

**Objective:**

The aim of this study was to develop an EHR-based pragmatic cardio-oncology registry and, as proof of principle, to investigate care gaps in the cardiovascular care of cancer patients.

**Methods:**

We generated a programmatically deidentified, real-time EHR-based cardio-oncology registry from all patients in our institutional Cancer Population Registry (N=8275, 2011-2017). We investigated: (1) left ventricular ejection fraction (LVEF) assessment before and after treatment with potentially cardiotoxic agents; and (2) guideline-directed medical therapy (GDMT) for left ventricular dysfunction (LVD), defined as LVEF<50%, and symptomatic heart failure with reduced LVEF (HFrEF), defined as LVEF<50% and Problem List documentation of systolic congestive heart failure or dilated cardiomyopathy.

**Results:**

Rapid development of an EHR-based cardio-oncology registry was feasible. Identification of tests and outcomes was similar using the EHR-based cardio-oncology registry and manual chart abstraction (100% sensitivity and 83% specificity for LVD). LVEF was documented prior to initiation of cancer therapy in 19.8% of patients. Prevalence of postchemotherapy LVD and HFrEF was relatively low (9.4% and 2.5%, respectively). Among patients with postchemotherapy LVD or HFrEF, those referred to cardiology had a significantly higher prescription rate of a GDMT.

**Conclusions:**

EHR data can efficiently populate a real-time, pragmatic cardio-oncology registry as a byproduct of clinical care for health care delivery investigations.

## Introduction

The success of cancer therapies has led to a growing population of cancer survivors, over 17 million in the United States in 2020. Surviving cancer no longer marks the final treatment goal but rather the beginning of “cancer survivorship.” An important facet of this care is the recognition and management of the cardiotoxic effects of cancer therapies, which include traditional metabolic diseases such as hypertension, dyslipidemia, and insulin resistance, as well as overt cardiovascular diseases, including coronary artery disease, left ventricular dysfunction (LVD), and heart failure with reduced left ventricular ejection fraction (HFrEF) [1]. Cardio-oncology has emerged as an important multidisciplinary specialty to provide cardiovascular care to the cancer patient. Practice guidelines from the American Society of Oncology (ASCO) and the European Society of Cardiology (ESC) provide specific recommendations such as left ventricular ejection fraction (LVEF) measurement assessment before and after treatment with potentially cardiotoxic agents such as anthracyclines and epidermal growth factor receptor 2 (HER2) blocking antibodies [2,3].

Electronic health records (EHRs) used for day-to-day patient care activities provide a unique repository of aggregate data about this at-risk population [4]. Hierarchical EHR databases harbor rich clinical data with specificity exceeding the information available from flat file claims data because EHR diagnoses are encoded with SNOMED CT (formerly Systematized Nomenclature of Medicine-Clinical Terms) instead of claims data that are encoded solely based on International Classification of Diseases, Tenth Revision (ICD-10) codes [5]. For instance, renal cell carcinoma, nephroblastoma, renal sarcoma, and multiple other kidney cancer types all share a single ICD-10 code and cannot be differentiated by ICD-10–encoded claims data, necessitating manual chart review for differentiation. EHR data are also accumulated in real time, rather than after a delay for claims submission and processing. These novel information management technologies can handle large-scale health care data more efficiently than traditional approaches for standard registries, which are massive endeavors.

In this study, we aimed to test the hypothesis of the feasibility to rapidly construct a cardio-oncology registry from existing EHR data and to employ such a registry, as proof of concept for (a) care gap identification for optimizing individual patient care, (b) analysis of one’s local population or local oncology management patterns, and (c) comparison of the use of guideline-directed medical therapy (GDMT) among patients who were referred to cardiology vs those who were not.

## Methods

### Study Population

Documentation of clinical care delivered to all patients at University of Texas Southwestern Health System is recorded within our enterprise-wide EHR, Epic (Epic Systems). Our overall EHR-based cancer population registry included all patients with a cancer diagnosis listed on their Problem List, using the intentionally broad SNOMED CT concept hierarchy-based value set definition: [Malignant neoplastic disease (disorder) (363346000), including descendants OR Carcinoma in situ (disorder) (109355002), including descendants OR Adenocarcinoma in situ in villous adenoma (disorder) (99741000119100), including descendants OR Neoplasm of brain (disorder) (126952004), including descendants] AND NOT [Benign neoplasm of brain (disorder) (92030004), including descendants OR Family history of clinical finding (situation) (416471007), including descendants]. A patient with any diagnosis on their Problem List that fit the above rule was included in the broad Cancer Population Registry (N=73,067).

**Figure 1:**
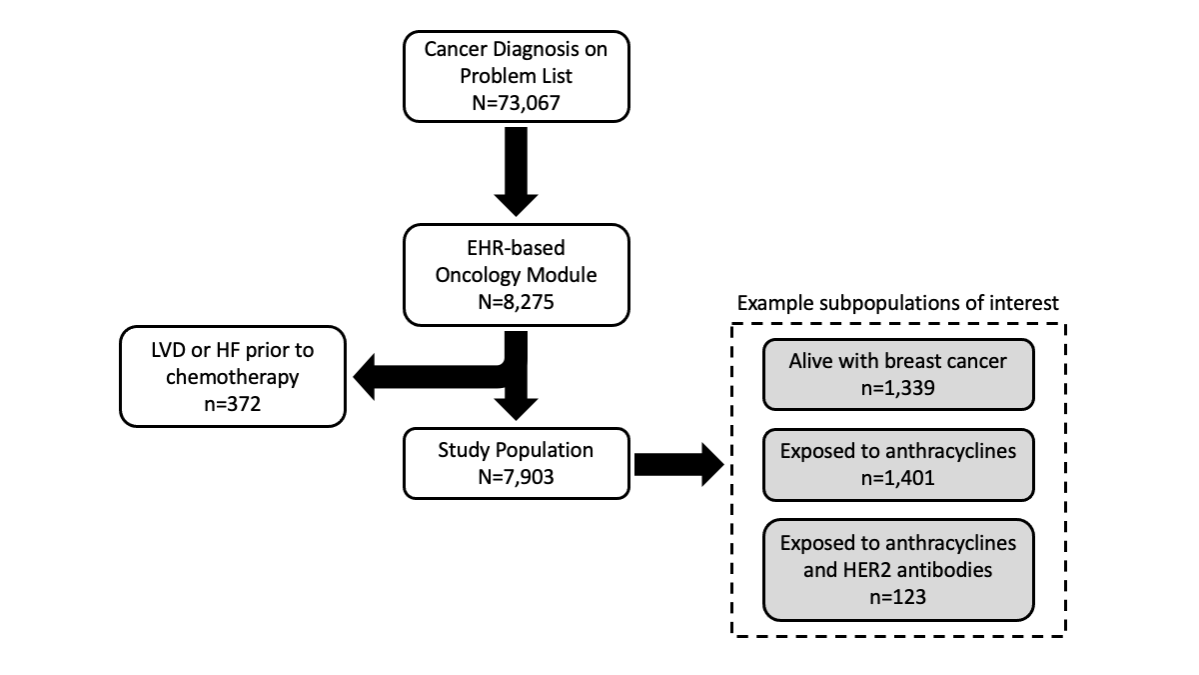
CONSORT diagram of patient populations. Our overall EHR-based cancer population registry includes all patients with a cancer diagnosis listed on their Problem List, using the intentionally broad SNOMED CT (Systematized Nomenclature of Medicine-Clinical Terms) concept hierarchy-based value set definition. A patient with any diagnosis on their Problem List that fits the above rule is included in the broad Cancer Population Registry (N=73,067). LVD: left ventricular dysfunction; HF: heart failure with reduced ejection fraction; EHR: electronic health record; HER2: epidermal growth factor receptor 2.

After filtering for patients with documentation in the EHR oncology module, the Cardio-oncology Registry members comprised 8275 patients who had received cancer treatment from January 1, 2011 until June 30, 2017. Patients meeting the criteria for LVD or HFrEF (see definitions below) that predated cancer treatment were excluded (n=372), leaving a final population of 7903 patients (Figure 1). More specific registry populations were then derived by filtering this broad registry by one or more criteria. Use of patient-level EHR data to construct this cardio-oncology registry was approved by the institutional review board at University of Texas Southwestern Medical Center. Registry development and data management of the EHR are further described in Multimedia Appendix 1 and the interface for registry population management is shown in Multimedia Appendix 2.

### Care Measures

For all cancer patients in the registry, if an LVEF measure was available prior to the chemotherapy initial exposure date, the patient was counted as having a prechemotherapy ejection fraction assessment. If any LVEF measure was available following their chemotherapy initial exposure date, they were counted as having a postchemotherapy LVEF assessment. For the purposes of this analysis, postchemotherapy LVD was defined as LVEF<50% (by any of the imaging modalities described previously) and postchemotherapy systolic HFrEF was defined as presence of systolic heart failure and/or dilated cardiomyopathy on the Problem List. Patients meeting the criteria for LVD or HFrEF prior to the chemotherapy initial exposure date were excluded (n=372). Manual chart review was performed by two authors (AC and VZ) to verify the diagnostic accuracy of the above-described methodology for data extraction. For LVD (ie, LVEF<50%) as detected by the EHR registry, manual review was performed on 100 randomly selected charts, including 50 charts of patients identified as having LVD measures and 50 charts of patients identified as not having LVD measures. Similarly, for HFrEF as detected by the EHR registry, manual review was performed on 200 randomly selected charts, including 100 charts of patients identified as having HFrEF measures and 100 charts identified for patients as not having HFrEF measures. Interrater agreements between the two authors were 86% for HFrEF and 94% for LVD. A third author (SD) adjudicated the cases in which AC and VZ disagreed and made the final decision.

Once posttreatment cardiac dysfunction has developed, neuro-hormonal medical therapy (beta-blockers, angiotensin-converting enzyme [ACE] inhibitors, mineralocorticoid receptor antagonists [MRA]) for cardiomyopathy with reduced ejection fraction is recommended according to American College of Cardiology (ACC)/American Heart Association (AHA) guidelines [6]. Registry patients with postchemotherapy LVD or HFrEF were considered to be on one of the GDMT drugs if the active medication list at the time of data extraction contained the following: (1) beta-blockers for HFrEF, including all formulations of carvedilol, metoprolol succinate, and bisoprolol; (2) ACE inhibitor, including all formulations with the First Data Bank (FDB) Pharmaceutical Class or Pharmaceutical Subclass title containing the phrase “ACE Inhibitor” (2 Pharmaceutical Classes plus 7 Pharmaceutical Subclasses), or all formulations of 8 combination medications containing an ACE inhibitor; (3) angiotensin receptor blocker (ARB), all formulations with the FDB Pharmaceutical Class title containing the phrase “Angiotensin Receptor Antagonist,” “Angiotensin Receptor Blocker,” Angiotensin II Receptor Blocker,” or abbreviations of these (6 Pharmaceutical Classes in total), along with all formulations of aliskiren/valsartan; (4) the patient was included as receiving “ACE-inhibitor/ARB” if their active medication list included either an ACE inhibitor, ARB, or both; and (5) MRA, including all formulations with FDB Pharmaceutical Subclasses containing the phrase “Aldosterone Receptor Antagonist” (2 Pharmaceutical Subclasses).

### Statistical Analysis

Categorical variables are shown as numbers and percentages. Continuous variables are shown as median (IQR). Comparisons between two dichotomous categorical variables were performed using the χ^2^ test. Sensitivity of EHR-based outcomes detection was calculated by dividing the number of true positive outcomes confirmed by manual chart review over the sum of true positives and false negatives. Specificity was calculated by dividing the number of true negatives over the sum of true negatives and false positives. Two-sided *P* values <.05 were considered significant. Statistical analysis was carried out using Microsoft Excel 365.

## Results

### Study Population

Among the 8275 patients included in our EHR cardio-oncology registry (Figure 1), the majority were women (Table 1). Their median age was 63 years. Over a quarter of the patients had hypertension and approximately 15% had diabetes. Their median BMI was 26 kg/m^2^. The most common treatment was anthracyclines, followed by HER2 antibodies and other tyrosine kinase inhibitors.

**Table 1:** Electronic health records–based cardio-oncology registry patient demographics and their clinical characteristics (N=8275).

Characteristic		Value
Age at time of data extraction (years), median (IQR)		63 (52-71)
Female gender, n (%)		4516 (54.57)
Alive at time of data extraction, n (%)		5576 (67.38)
Hypertension, n (%)		2135 (25.80)
Diabetes mellitus, n (%)		1013 (15.24)
BMI (kg/m^2^), median (IQR)		26 (23-30)
Systolic blood pressure (mmHg), median (IQR)		120 (96-135)
Diastolic blood pressure (mmHg), median (IQR)		73 (53-78)
Breast cancer, n (%)		1585 (19.15)
Time from Beacon chemotherapy (days), median (IQR)		431 (200-689)
**Cancer treatment, n (%)**		
	Anthracyclines	1472 (17.78)
	HER2^a^ antibodies	410 (4.95)
	Tyrosine kinase inhibitors	730 (8.82)
	Immune checkpoint inhibitors	26 (0.31)
**Prechemotherapy LVEF^b^ assessment method, n (%)**		
	Echocardiogram	1597 (19.29)
	MUGA^c^ scan	25 (0.30)
	Cardiac MRI^d^	16 (0.19)
	None	6637 (80.2)
**Postchemotherapy LVEF assessment method** **, n (%)**		
	Echocardiogram	3362 (40.62)
	MUGA scan	17 (0.21)
	Cardiac MRI	13 (0.15)
	None	4883 (59.01)

^a^HER2: human epidermal growth factor receptor 2.

^b^LVEF: left ventricular ejection fraction.

^c^MUGA: multigated acquisition scan.

^d^MRI: magnetic resonance imaging.

### Care Measures

**Figure 2:**
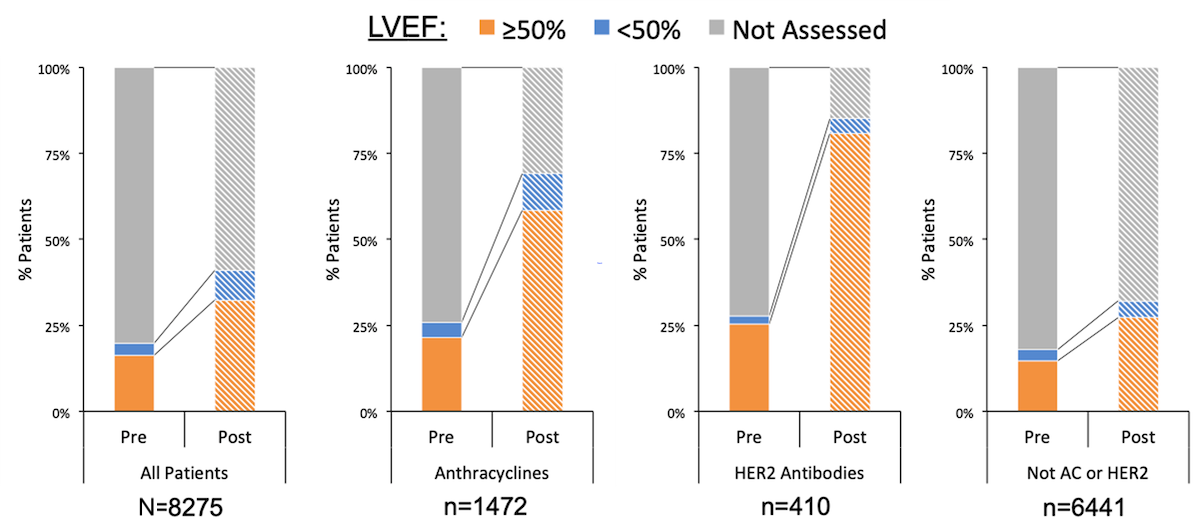
Documented LVEF assessment before and after cancer therapy. LVEF: left ventricular ejection fraction; HER2: human epidermal growth factor receptor 2 blocking antibodies; AC: anthracyclines.

LVEF was documented prior to initiation of chemotherapy in 1636 (19.77%) of all 8275 patients (Figure 2), with 97.5% of these patients having echocardiogram as the LVEF assessment method. Documented prechemotherapy LVEF assessment did not vary significantly by chemotherapy categories such as anthracyclines or HER2 antibodies (25.88% vs 27.8%, *P*=.43). After the chemotherapy initial treatment date, a significantly higher percentage of all patients had a documented ejection fraction assessment compared to assessment prior to chemotherapy initiation (3385/8275, 40.91% vs 1636/8275, 19.77%; *P*<.001). Patients treated with anthracyclines or HER2 antibodies had a significantly higher frequency of postchemotherapy LVEF assessment than patients not exposed to these known cardiotoxic therapies (69.1% vs 32% and 85.1% vs 32%, respectively; *P*<.001 for both comparisons).

### Comparison of Registry-Based Outcomes Detection Strategies

To validate the performance of an EHR-based cardio-oncology registry to identify clinically relevant outcomes, a random selection of patient charts (n=300, 100 for LVD and 200 for HFrEF) were reviewed for diagnostic accuracy. For detection of postchemotherapy LVD (LVEF<50%), only the patients undergoing postexposure screening (n=3196) were considered to be at risk. Compared with manual review of all cardiovascular imaging study reports, the SQL query–based method of LVEF extraction had 100% sensitivity and 83% specificity for detection of LVD (Table 2). For detection of postchemotherapy systolic heart failure, all patients completing cancer treatment at our institution (N=7903) were considered to be at risk. Compared with manual chart review for indicators of systolic heart failure (Problem List, echocardiogram, N-terminal pro b-type natriuretic peptide, discharge diagnoses), the Problem List method had 95.3% sensitivity and 83.5% specificity for detection of HFrEF.

**Table 2:** Validation of electronic health records (EHR)-based outcomes detection.

Performance metric	Left ventricular dysfunction	HFrEF^a^
EHR definition	EF^b^<50%	Problem List entry
Charts reviewed, n	100	200
Population at risk (n)	Surveillance echo (3196)	Completed chemotherapy (7903)
Sensitivity, % (95% CI)	100 (91.2-100)	95.3 (88.4-98.7)
Specificity, % (95% CI)	83 (71.5-91.7)	83.5 (75.4-89.8)
Positive predictive value, % (95% CI)	80 (69.4-87.6)	81 (73.8-86.6)
Negative predictive value, % (95% CI)	100 (N/A^c^)	96 (90.2-98.4)

^a^HFrEF: heart failure with reduced left ventricular ejection fraction.

^b^EF: ejection fraction.

^c^N/A: not applicable.

### Prevalence of Postcancer Treatment Cardiomyopathy

Overall, the prevalence of LVD (LVEF<50%) among the subpopulation with postchemotherapy LVEF assessment was 9.4% (Table 3). This prevalence rate varied by treatment exposure, with patients receiving anthracyclines and HER2 antibodies having lower incidences compared with those of other types of chemotherapy. In a sensitivity analysis, we selected patients who had their LVEF assessed prior to chemotherapy and then had their LVEF assessed after chemotherapy (n=833). Of those, 117 patients (13.7%) were found to have LVD, which is higher when compared with the rate of 9.4% reported above.

In contrast to LVD (which was captured by SQL queries of cardiovascular procedures), HFrEF is a clinical entity that can be captured by Problem List documentation. The overall prevalence of documented postchemotherapy HFrEF was 2.5%. Patients exposed to HER2 antibodies had a significantly higher prevalence (*P*<.001) compared with that of all other patients (Table 3).

**Table 3:** Prevalence of left ventricular (LV) dysfunction and heart failure with reduced left ventricular ejection fraction (HFrEF) following chemotherapy.

Chemotherapy	LVEF^a^ assessed, N	LV dysfunction, n (%)	Problem List documented, N	HFrEF, n (%)
All	3196	299 (9.4)	7903	202 (2.5)
Anthracyclines	979	41 (4.2)	1401	50 (3.5)
HER2^b^ antibodies	322	16 (4.9)	399	36 (9.0)
Not AC^c^/HER2	1923	243 (12.6)	6151	122 (1.9)

^a^LVEF: left ventricular ejection fraction.

^b^HER2: human epidermal growth factor receptor 2.

^c^AC: anthracyclines.

### Care Gap Identification: Use of GDMT in Patients With Postcancer Treatment Cardiomyopathy

Of the patients who developed postchemotherapy LVD or HFrEF, 237 (63.9%) were referred to cardiology. Compared with patients who were not referred to cardiology, those who were referred to cardiology had a significantly higher frequency of prescriptions for beta-blockers (44.1% vs 18.8%, *P*<.001), ACE inhibitors/ARBs (47.4% vs 30.6%, *P*=.01), and MRA (11.8% vs 2.4%, *P*=.01).

## Discussion

### Principal Findings

In this study, we have demonstrated that rapid development of an EHR-based cardio-oncology registry is feasible and yields actionable information early, with a performance in identifying clinically relevant outcomes very similar to that of manual chart abstraction. In a proof-of-concept application, we identified that: (1) baseline LVEF prior to initiation of cancer therapy was documented in only 20% of patients treated for cancer; (2) the prevalence of LVD and HFrEF related to cancer therapeutics was relatively low (9.4% and 2.5%, respectively); and (3) among patients who developed postchemotherapy LVD or HFrEF, those who were referred to cardiology had significantly more prescriptions for a GDMT.

Clinical guidelines in this field are relatively new [2,3], creating opportunities for identifying and closing care gaps through population health–based approaches, with the goal of enhancing patients’ long-term outcomes. Pragmatic registries using EHR data collected as a byproduct of clinical care would prove more practical than manual chart abstraction for scaling to meet local and national needs [7]. The ability of an EHR-based cardio-oncology registry to identify care gaps in real time could help identify patients not meeting guideline-directed cardiotoxicity surveillance timelines. We discovered that only a minority of the patients treated for cancer at our institution had a documented baseline LVEF measure prior to initiation of cancer therapy. Although uniform echocardiographic prescreening of all cancer patients is not indicated or cost-effective, this screening pattern is inadequate and likely leads to underestimation of prechemotherapy cardiovascular risk. It is worth noting that if our patients had received an LVEF assessment at another facility, they would not have been captured in our analysis. Thus, it is likely that we underestimated the prevalence of LVEF assessment pre and postchemotherapy. Significantly more patients had an LVEF assessment after the oncology treatment start date, with patients receiving HER2 antibodies having the highest rate of echocardiographic assessment. This observed difference in postexposure screening is in line with the established structural cardiotoxicity of HER2 antagonism and the Food and Drug Administration–recommended screening interval of every 3 months [8]. Interestingly, our study showed that patients exposed to nonanthracyclines and non-HER2 targeted chemotherapies were significantly less likely to undergo postexposure echocardiography. We suspect that this is due to underrecognition of the potential cardiotoxicity of the other widely used chemotherapeutic agents. Additionally, practice guidelines regarding LVEF assessment before and after treatment with potentially cardiotoxic agents were not available for most of the time period covered in this study (January 1, 2011 to June 30, 2017) as the ESC and the ASCO guidelines were released in 2016 and 2017, respectively.

An EHR-based cardio-oncology registry can also provide descriptive statistics on the local oncology population as a byproduct of routine clinical care. Overall, incidences of LVD and HFrEF postcancer treatment were low (9.4% and 2.5%, respectively). Of note, patients receiving anthracyclines and HER2 antibodies had a lower incidence of LVD when compared with that of patients receiving other types of chemotherapy. This difference likely reflects selection bias and relative underscreening of the population exposed to chemotherapy classes not traditionally viewed as cardiotoxic. In contrast to LVD (which was captured by SQL queries of cardiovascular procedures), HFrEF is a clinical entity that can be captured by Problem List documentation. Patients exposed to HER2 antibodies had a significantly higher prevalence of HFrEF documentation. This is perhaps attributable to increased provider awareness of this medication’s cardiotoxic effects and a tendency to code volume overload states in these patients as heart failure. These prevalence estimates varied based on chemotherapy exposure but are also likely influenced by cancer type, heterogeneity of echocardiographic screening, as well as referral bias. Nevertheless, the observational data derived from routine clinical care provide an opportunity for narrowing the focus of pre and postexposure screening efforts. We are currently investigating use of this EHR registry to develop a predictive tool to estimate the risk of cancer therapeutics–related cardiac dysfunction at the time of cancer diagnosis.

Once posttreatment cardiac dysfunction has developed, neurohormonal GDMT for cardiomyopathy with a reduced ejection fraction is recommended according to ACC/AHA guidelines [6]. Perhaps of most interest and reflective of other “real-world” heart failure experiences such as the CHAMP-HF registry, we found that adherence to guideline-directed medical therapies was suboptimal in patients with cardiomyopathy following chemotherapy [9]. It is unclear whether this reflects the general underutilization of GDMT in ambulatory HFrEF patients or an undertreatment phenomenon when cancer and HFrEF coexist. Referral to a cardiologist was associated with significant improvement in guideline-recommended beta-blocker, ACE inhibitor/ARB, and MRA prescriptions. Our relatively low prevalence of GDMT use is likely due to HFrEF being defined as LVEF<50% in our registry rather than the threshold of <40% used in other registries or clinical trials. We were also unable to assess contraindications to GDMT such as hypotension or hyperkalemia.

Our study further supports the premise that pragmatic clinical research employing EHR data can be feasible and fruitful [10-14]. EHR-based registries for specialized conditions can be constructed in short time frames (weeks to months) using replicable frameworks [4] and can then be employed for investigation. For multisite, multi-EHR studies, mapping of EHR fields to standard terminologies (SNOMED, LOINC, RxNorm) now required for EHR certification on interoperability can be leveraged for defining conditions [5], observations, and medications identically across all sites. Multicenter studies are expedited by adoption of a common data model. In the future, writing SQL code once for transforming each of the large EHR vendors’ data models to the common data model, and then sharing the transformed SQL code scripts among each vendors’ customers, would greatly facilitate multiinstitution, multi-EHR clinical research. Clinical imaging data increasingly extends the range of digitized patient information useful for analytics and clinical research [15]. Applying machine learning and other forms of artificial intelligence to analyze the information contained within the images themselves will increasingly add important insights [16]; this field is poised for further major advances.

Developing an institution-wide cardio-oncology registry, as we have done here, enables local care gap closure initiatives and can foster future clinical research projects. Moreover, combining experiences across multiple institutions offers the promise of advancing the field faster, and with broader applicability and patient benefit. As above, adopting standard terminologies (mapping local EHR codes to standard codes) greatly facilitates combining data from multiple sites. Additionally, the use of standard Health Level Seven International Fast Healthcare Interoperability Resources (FHIR) now enables communication of data between EHRs and a common registry database. For instance, we have successfully employed FHIR to integrate data from our produced Epic EHR to a REDCap study database [17]. Thus, one can envision a national/international REDCap cardio-oncology registry database—either a single shared database or a federated database employing a common structure—that is able to receive contributions from multiple sites via FHIR-enabled EHR connections. Such a structure would streamline the acquisition and curation of EHR-derived registry data from multiple sites on diverse EHRs.

### Limitations

For this initial report, we used a single cancer treatment start date, corresponding to the patient’s first treatment episode on our EHR’s oncology module. Some patients can have more than one cancer, or a late recurrence of an original cancer, and thus have more than one cancer treatment episode. More sophisticated analyses would require performing some evaluations at the episode level rather than the patient level and including start/stop dates of treatments. Chemotherapy dosing is not accounted for in this analysis; thus, more sophisticated dose-effect or epidemiologic studies would also be needed to account for this variable. For all of the above, the additional data elements needed are collected in the EHR as a byproduct of clinical care, and such additionally requested information types can be added iteratively to the Cancer Population Registry, expanding relevance and utility for multiple purposes.

We used each patient’s Problem List as the source of their oncology diagnoses as well as their comorbid conditions. Although Problem List completeness remains an area of concern for pragmatic clinical trials and registries [18], Problem List diagnoses prove to be more specific than encounter or claims diagnoses, since the latter are allowed to be used to indicate “rule-out” conditions [19]. In our setting, Problem List diagnoses were used for cancer staging in our EHR oncology module and for linking Oncology Treatment Plans and Episodes to diagnoses, both of which tended to ensure the presence of active cancer diagnoses. Patients also received a copy of their Problem List at each visit and on their patient portal for coverification. In the future, we are planning to increase our use of clinical decision support systems and automated additions as studies have shown that these can enhance Problem List completeness [20-23].

### Conclusions

Cardiac complications of both established and newer chemotherapy agents have given rise to the emerging subspecialty field of cardio-oncology and generated guidelines for optimizing care. EHR-derived population health tools for detecting and resolving care gaps are needed. From this EHR-based cardio-oncology registry, we found (a) an apparent care gap in adherence to guidelines for baseline ejection fraction assessment; (b) documented postchemotherapy cardiac dysfunction to be a relatively rare event; and (c) a second care gap in prescribing guideline-directed medications for patients with posttreatment cardiomyopathy, with improved rates among patients seen by a cardiologist. As a byproduct of clinical care, EHR data can efficiently populate a real-time pragmatic registry of cardio-oncology patients with data enabling pragmatic comparative effectiveness research.

## Appendix

Multimedia Appendix 1 Registry development, data collected from electronic health records (EHRs), and data management.

Multimedia Appendix 2 Screenshot of registry population management user interface. Patient data were displayed within the electronic health record’s population health module.

## Abbreviations

ACC: American College of Cardiology
ACE: angiotensin converting enzyme
AHA: American Heart Association
ARB: angiotensin receptor blocker
ASCO: American Society of Oncology
EHR: electronic health record
ESC: European Society of Cardiology
FDB: First Data Bank
FHIR: Fast Healthcare Interoperability Resources
GDMT: guideline-directed medical therapy
HER2: human epidermal growth factor receptor 2
HFrEF: heart failure with reduced left ventricular ejection fraction
ICD-10: International Classification of Diseases, Tenth Revision
LVD: left ventricular dysfunction
LVEF: left ventricular ejection fraction
MRA: mineralocorticoid receptor antagonist
