# Supplementary material for: Electronic Health Records–Based Cardio-Oncology Registry for Care Gap Identification and Pragmatic Research: Procedure and Observational Study
Source: JMIR Cardio. 2021 May 12;5(1):e22296. doi: 10.2196/22296 (PMC8411429; doi:10.2196/22296)
Supplement: Multimedia Appendix 1 [file cardio_v5i1e22296_app1.doc]

### Registry Development

The cardio-oncology registry and its features were developed in the EHR during 2-week iterations, using a previously described agile approach for multiple specialty registry development.Error: Reference source not found EHR-collected data elements added included cancer types, chemotherapy medication classes (anthracyclines, immune checkpoint inhibitors, tyrosine kinase inhibitors, HER2 antagonists/antibodies), cardiovascular medication classes [beta-blockers, angiotensin cardioverting enzyme (ACE-inhibitors), angiotensin receptor blocker (ARB), mineralocorticoid receptor antagonists (MRA)], cardiovascular procedures for assessment of LVEF, cardiac diagnoses (heart failure, coronary disease, atrial fibrillation), and cardiac risk factors such as diabetes, hypertension, lipid levels, smoking status and body mass index (BMI) (**Supplemental Table 1**).

**Supplemental Table 1.** EHR-collected clinical data elements for cardio-oncology registry

| **Clinical Data** | **Examples** | **EHR****^a^ Source** | **Classification Method** |
| --- | --- | --- | --- |
| Cancer Type | Lung cancer, breast cancer, colon cancer | Patient’s list of medical conditions (Problem List) | SNOMED CT concept hierarchy-based value sets (diagnosis groupers) |
| Chemotherapy Medication Classes | Anthracyclines, checkpoint inhibitors, protein kinase inhibitors,  **^b^**HER2 blockers | Patient’s history of all Medication Orders | Pharmaceutical class and sub-class (supplied by drug database from **^c^**FDB) |
| Cardiovascular Medications | **^d^**ACE-inhibitor, **^e^**ARB, beta-blockers for heart failure, **^f^**MRA | Patient’s current Medication List | Pharmaceutical class and sub-class (supplied by drug database from FDB) |
| Cardiac Risk Factors: Diagnoses | Diabetes mellitus, hypertension | Patient’s list of medical conditions (Problem List) | SNOMED CT concept hierarchy-based value sets (diagnosis groupers) |
| Cardiac Risk Factors: Other | Smoking status, lipid levels, body mass index | Social history, laboratory results, vital sign flowsheet | N/A (EHR-entered values used directly) |

**^a^**EHR: electronic health records.

**^b^**HER2– epidermal growth factor receptor 2.

**^c^**FDB – First Data Bank.

**^d^**ACE – angiotensin converting enzyme.

**^e^**ARB – angiotensin receptor blocker.

**^f^**MRA – mineralocorticoid receptor antagonist.

Medical conditions were defined using SNOMED CT concept hierarchy-based (“intensional”) value set rules for simplicity and reproducibility across EHRs [5]. Patient data were displayed within the EHR’s population health module, which supports care gap identification and outreach for care gap closure (**Supplemental Figure 1**).

### Data Management

De-identified data were exported from the population health reporting module (Reporting Workbench) and from the EHR’s companion relational reporting database module (Clarity) into our data warehouse business intelligence platform (Microsoft SQL Server, Power BI) for data integration and visualization. Cardiovascular imaging reports were retrieved from Clarity, from which EF values were then extracted using Structured Query Language (SQL) queries. Cardiac magnetic resonance imaging (MRI) and multi-gated acquisition scan (MUGA) studies returned a single LVEF value per report. Echocardiogram reports contained multiple LVEF values; when present, the highest prioritized LVEF value was chosen, using the following prioritization order, from highest to lowest: biplane method-of-discs, single plane method-of-discs 2-chamber view, single plane method-of-discs 4-chamber view, Teichholz method.

Chemotherapy initial exposure dates were determined by examining each patient’s treatment plan episodes recorded in the EHR oncology module (Beacon). Treatment plans containing chemotherapy were identified, and the start date of the first such treatment plan used as the initial treatment date. Patient encounters with cardiologists were identified using E&M codes from professional billing data, also stored in the EHR and retrieved via Clarity. At our institution, both inpatient and outpatient professional billing is stored in the single EHR instance. Accordingly, for each patient, any CPT codes billed by cardiologists for Office Visits, Hospital E&M Encounters (admissions, subsequent hospital visits) and Consultations (inpatient and outpatient) were considered to represent a patient encounter with a cardiologist.

These datasets were combined to yield a table with one row per patient, with the following data points as columns:

- Chemotherapy initial exposure date (derived from Beacon treatment episode date, as above)
- Last LVEF prior to chemotherapy exposure date: date of measurement
- Last LVEF prior to chemotherapy exposure date: LVEF value
- First LVEF after chemotherapy exposure date: date of measurement
- First LVEF after chemotherapy exposure date: LVEF value
- Last cardiologist encounter date prior to chemotherapy exposure date
- First cardiologist encounter date after chemotherapy exposure date

Interactive analyses were performed using Microsoft Power BI dashboards and Excel pivot tables as front-end tools. This project employed: our existing EHR clinical documentation, reporting, and population health modules (all from Epic Systems, Verona, WI), and our existing enterprise data warehouse (EDW) and business intelligence (BI) tools: Microsoft SQL Server, SQL Server Analysis Services, and Power BI (all from Microsoft, Redmond, WA).
