# Supplementary figures and images for: Electronic Health Records–Based Cardio-Oncology Registry for Care Gap Identification and Pragmatic Research: Procedure and Observational Study
Source: JMIR Cardio. 2021 May 12;5(1):e22296. doi: 10.2196/22296 (PMC8411429; doi:10.2196/22296)

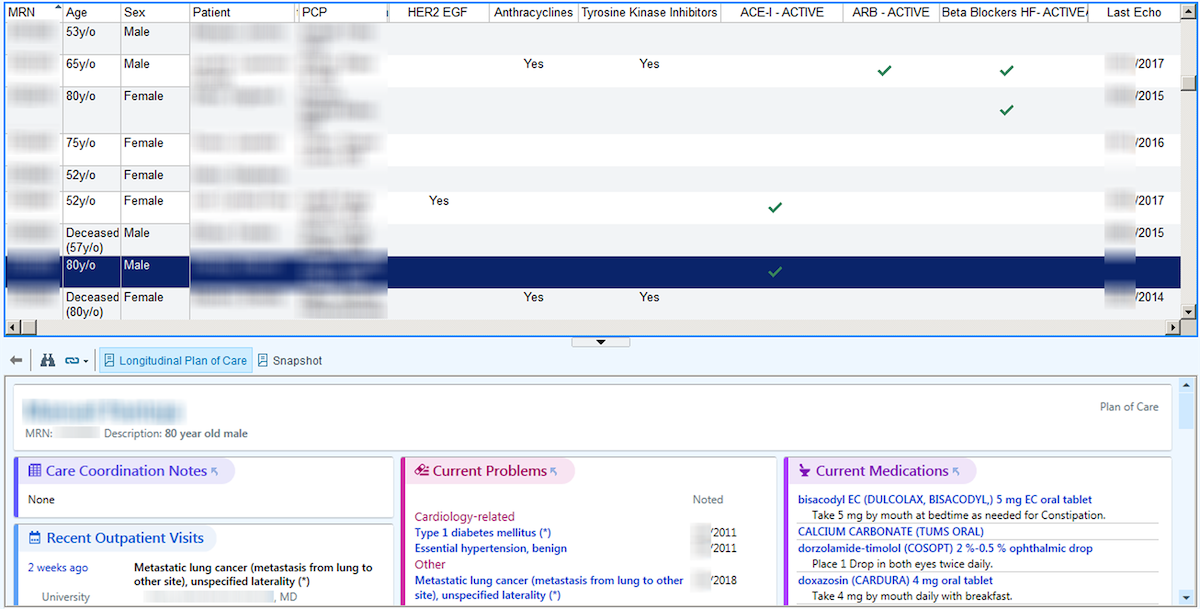

Supplement: Multimedia Appendix 2 [file cardio_v5i1e22296_app2.png]
